# Beta-asarone, a major component of Acorus tatarinowii Schott, attenuates focal cerebral ischemia induced by middle cerebral artery occlusion in rats

**DOI:** 10.1186/1472-6882-13-236

**Published:** 2013-09-25

**Authors:** Yuan-Xiao Yang, Yi-Tao Chen, Xiao-Jie Zhou, Chun-Lan Hong, Chang-Yu Li, Jian-You Guo

**Affiliations:** 1 Zhejiang Chinese Medical University, Hangzhou 310053, PR China; 2 Key Laboratory of Mental Health, Institute of Psychology, Chinese Academy of Sciences, Beijing 100101, PR China

## Abstract

**Background:**

Ischemic hypoxic brain injury often causes irreversible brain damage. The lack of effective and widely applicable pharmacological treatments for ischemic stroke patients may explain a growing interest in traditional medicines. β-Asarone, which has significant pharmacological effects on the central nervous system (CNS), was used in the prevention of cerebral ischemia in this paper.

**Methods:**

The right middle cerebral artery occlusion model was used in the study. The effects of β-Asarone on mortality rate, neurobehavior, grip strength, lactate dehydrogenase, glutathione content, Lipid peroxidation, glutathione peroxidase activity, glutathione reductase activity, catalase activity, Na^+^-K^+^-ATPase activity and glutathione S transferase activity in a rat model were studied respectively.

**Results:**

β-Asarone significantly improved the neurological outcome after cerebral ischemia and reperfusion in terms of neurobehavioral function in rats. Meanwhile, supplementation of β-Asarone significantly boosted the defense mechanism against cerebral ischemia via increasing antioxidants activity related to lesion pathogenesis. Restoration of the antioxidant homeostasis in the brain after reperfusion may help the brain recover from ischemic injury.

**Conclusions:**

These experimental results suggest that complement β-Asarone is protective against cerebral ischemia in specific way. The administration of β-Asarone could reduce focal cerebral ischemic/reperfusion injury. The Mechanism of β-Asarone in protection of cerebral ischemia was via increasing antioxidants activity related to lesion pathogenesis.

## Background

Ischemic hypoxic brain injury often causes irreversible brain damage. The cascade of events leading to neuronal injury and death in ischemia includes the release of cytokines and free radicals, and induction of inflammation, apoptosis, and excitotoxicity [1]. Reperfusion of ischemic areas could exacerbate ischemic brain damage through the generation of reactive oxygen species. The lack of effective and widely applicable pharmacological treatments for ischemic stroke patients may explain a growing interest in traditional medicines.

**Figure 1:**
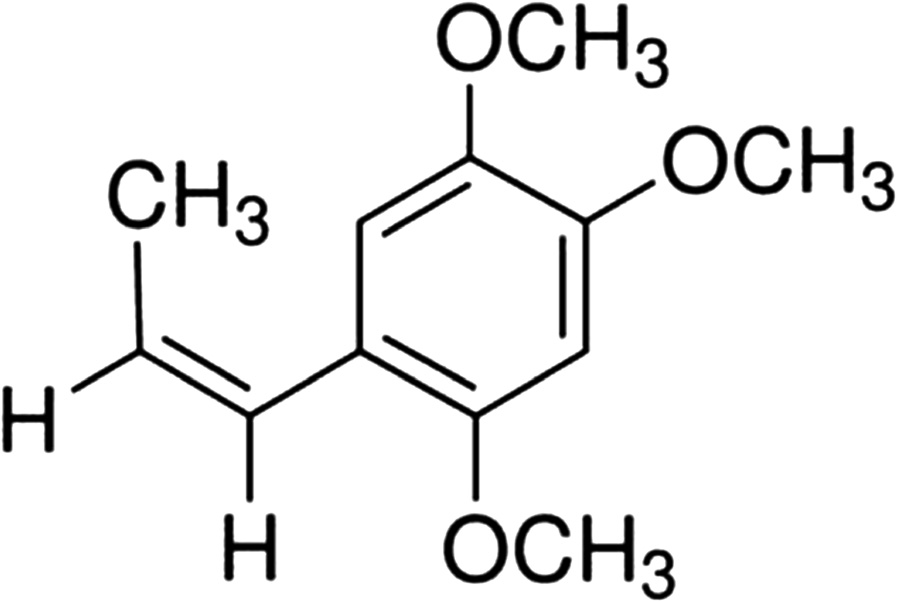
The chemical structure of β-Asarone.

Acorus tatarinowii Schott is native to Central Asia, North America and Eastern Europe [2]. β-Asarone (cis-2,4,5-trimethoxy-1-allyl phenyl), which can affect the central nervous system (CNS) [3-6], is a major component of Acorus tatarinowii Schott. β-Asarone could pass the blood–brain barrier (BBB) and thus enter the brain [7]. It has been reported that β-Asarone (Figure 1)could attenuate neuronal apoptosis in rat hippocampus and might be a potential candidate for development as a therapeutic agent to manage cognitive impairment associated with conditions such as Alzheimer’s disease [8,9]. Other authors found β-Asarone could reduce the toxicity of excitatory amino acids and increase the expression of c-fos in the epileptic rat brain [10]. In addition, β-Asarone could reduce the injuries of blood vessel endothelium and nerve cells of the cortex [11] and improve the cognitive function of the beta-amyloid hippocampus injection rats [12].

Thus, we hypothesize that β-Asarone possess protective effect of against ischemia-induced brain infarction. The present study was aimed to investigate the effects of β-Asarone on mortality rate, neurobehavior, grip strength, lactate dehydrogenase, glutathione content, Lipid Peroxidation, glutathione peroxidase activity, glutathione reductase activity, catalase activity, Na^+^-K^+^-ATPase activity and glutathione S transferase activity in a rat model.

## Methods

### Animals

Healthy adult Wistar rats (2 months old and weighing 225 ± 25 g) were used in the study. This study was approved by Zhejiang Chinese Medical University’s ethics committee, and all procedures complied with the guidance set out in the Guidelines for Caring for Experimental Animals published by the Ministry of Science and Technology of the People’s Republic of China. Every care was taken to minimize discomfort, distress, and pain.

### Chemicals

Acorus tatarinowii Schott were collected in Bozhou, China. Dr. Changyu Li at the Zhejiang Chinese Medical University established the identity of these species. β-Asarone was obtained as reported by Zanoli et al. [13]. Purity up to 99.55% was confirmed by gas chromatography–mass spectrometry (GC-MS), infrared spectrum (IR) and nuclear magnetic resonance (NMR) detection. The voucher specimen (No. SAC/20120412) was deposited at the Herbarium of the College of Pharmacology, Zhejiang Chinese Medical University, China.

### Experimental design

The animals were separated into five groups of ten rats each. The first group served as sham (SHAM). The second group was the ischemic group (MCAO). Group I and group II were treated orally by distilled water for 30 days respectively. Group III (β-Asarone−10), Group IV (β-Asarone−20) and Group V (β-Asarone−30) were treated orally by β-Asarone (10, 20 and 30 mg/kg/day, respectively) for 30 days followed by middle cerebral artery occlusion (MCAO) induced cerebral ischemia.

The right MCAO was performed using an intraluminal filament model and the method described by Liu et al. [14]. In brief, the rats were anesthetized with chloral hydrate (400 mg/kg, i.p.), a 4-0 nylon monofilament with a blunt end was introduced into the external carotid artery (ECA) and advanced into the middle cerebral artery via the internal carotid artery (ICA) (17-20 mm), until a slight resistance was felt. Successful occlusion was confirmed by an 87-90% reduction in cerebral blood flow (CBF), as measured by laser-Doppler flowmetry [15].

Two hours after the induction of ischemia, the filament was slowly withdrawn and the animals were returned to their cages for a period of 22 hours of reperfusion. Throughout the procedure, the body temperature was maintained at 37°C, with a thermostatically controlled infrared lamp. In sham rats, the ECA was surgically prepared for the insertion of the filament, but the filament was not inserted. The final number of rats was as follows: SHAM group n = 10; MCAO group n = 6; β-Asarone-10 group n = 8; β-Asarone−20 group n = 8 and β-Asarone−30 group n = 9.

### Neurobehavioral test

The sensorimotor integrity was conducted to assess the neurobehavior at 24 h after MCAO in rats [16]. Five categories of motor neurological findings were scored: 0, no observable deficit; 1, forelimb flexion; 2, forelimb flexion and decreased resistance to lateral push; 3, forelimb flexion, decreased resistance to lateral push and unilateral circling; 4, forelimb flexion, unable or difficult to ambulate. Animals that showed the features of the higher scores also showed all the features of the lower grades.

### Grip strength study

Grip strength in all the animals was measured for evaluation of neuromuscular strength, as described by Ali *et al*. [17]. The neuromuscular strength tests were carried out between 9:00 a.m. to 4:00 p.m. under standard laboratory conditions.

### Tissue preparation

After grip strength measurement, blood samples were drawn from the tail vein from all the groups and serum was separated for biochemical estimations. Thereafter, the animals were sacrificed immediately and their brains were taken out to dissect the hippocampus (HIP). Post-mitochondrial supernatant (PMS) obtained from 10% homogenate of tissue was used for the estimation of various parameters related with oxidative stress.

### Biochemical estimations

In serum, lactate dehydrogenase (LDH) was estimated using a method described by Khan *et al*. [18]. The PMS and HIP were used for the assay of glutathione (GSH) content, Lipid peroxidation (LPO), glutathione peroxidase (GPx) activity, glutathione reductase (GR) activity, catalase (CAT) activity, Na^+^-K^+^-ATPase activity and glutathione S transferase (GST) activity [19-24].

### Statistical analysis

The data are expressed as mean ± SEM. Statistical differences between means were determined by one-way analysis of variance (ANOVA), followed by Dunnett t-test. The values of *P* < 0.05 were considered as significant.

## Results and discussion

In this study, the cerebroprotective effect of β-Asarone on ischemic neuronal damage was clearly demonstrated using focal ischemia model rats.

The behavioral tasks adopted in this study were designed to assess impairments consistent with the known functional architecture of the rat brain.

**Figure 2:**
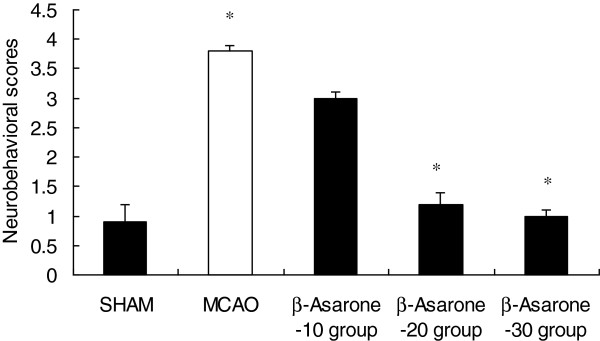
**Effect of β-Asarone on the development of behavioral abnormalities after middle cerebral artery occlusion.** Values are shown as means ± SEM. *p < 0.05 vs. MCAO group, **p < 0.01 vs. MCAO group. (F_SHAM_ =17.642, df_SHAM_=11, p_SHAM_=0.001, F_β-Asarone−10_=2.252, df_β-Asarone−10_=11, p_β-Asarone−10_=0.162, F_β-Asarone−20_=16.396, df_β-Asarone−20_=11, p_β-Asarone-20_ = 0.01, F_β-Asarone−30_=22.738, df_SHAM_=11, p_SHAM_=0.02).

Twenty-four hours after MCAO in rats, neurological deficit scores were significantly reduced in β-Asarone−20 -treated rats and β-Asarone−30-treated rats. The neurobehavior for the SHAM group was 0.9 (0.6-1.1), the MCAO group was 3.7 (2.6-5.3), the β-Asarone−10 group was 3.0 (2.2-4.1), the β-Asarone−20 group was 1.2 (1.0-4.1) and the β-Asarone−30 group was 1.0 (1.0-3.0). It is clear that the behavioral abnormality was significantly developed in the MCAO group as compared with the sham (Figure 2). In contrast, the β-Asarone−20 group and β-Asarone−30 group significantly suppressed the development of behavioral abnormality as compared with the MCAO group (Figure 2).

The grip strength in the SHAM group was found to be 0.888 ± 0.008 kg units. A significant decrease in the grip strength was observed in the MCAO group (0.506 ± 0.002), as compared to the sham rats (*P* < 0.01). Both β-Asarone−20 and β-Asarone−30 treated rats showed a significant increase in grip strength, as compared to the MCAO group (*P* < 0.01) (Table 1).

**Table 1:** Effect of β-Asarone on basal grip strength

**Different groups**	**Grip strength**	**F-values**	**df-values**	**p-values**
	**(Kg Units)**			
MCAO group	0.506 ± 0.002^b^			
SHAM group	0.888 ± 0.008^a^	0.155	11	0.002
β-Asarone-10 group	0.560 ± 0.022^b^	0.154	11	0.005
β-Asarone-20 group	0.788 ± 0.004^a^	0.145	11	0.001
β-Asarone-30 group	0.799 ± 0.020^a^	2.974	11	0.004

Values are shown as means ± SEM. The different letters in the same column indicate a statistical difference (*p* < 0.01 vs. MCAO group).

Increasing evidence has indicated that ischemia/reperfusion occurs due to oxidative stress that may potentiate ischemic injury [25]. Lactate dehydrogenase was measured to evaluate the role of antioxidative stress in the protection of β-Asarone. The serum LDH levels in SHAM group were found to be 80.200 ± 1.233 IU/L. A significant increase in the activity of LDH in serum was observed in MCAO group, as compared to the SHAM group; whereas, β-Asarone−20 and β-Asarone−30 treatment significantly resulted in decreased serum LDH levels when compared with MCAO group rats (Table 2).

**Table 2:** Effect of β-Asarone on serum LDH levels

**Different groups**	**LDH (IU/L)**	**F-values**	**df-values**	**p-values**
MCAO group	156.200 ± 3.331			
SHAM group	80.200 ± 1.233^***^	6.555	5.183	0.000
β-Asarone-10 group	166.600 ± 2.111	7.190	11	0.118
β-Asarone-20 group	126.630 ± 3.111^**^	7.490	5.063	0.001
β-Asarone-30 group	111.231 ± 2.111^**^	0.425	11	0.005

Values are shown as means ± SEM. **p <0.01 vs. MCAO group, ***p <0.001 vs. MCAO group.

Reduced glutathione (GSH) is one of the primary endogenous antioxidant defense systems in the brain, which removes hydrogen peroxide and lipid peroxides. Decline in GSH levels could either increase or reflect oxidative status [26,27]. Concentrations of GSH were lower in MCAO group than those in SHAM group (Tables 3 and 4). β-Asarone−20 treatment significantly increased the GSH.level. The same results did occur in the β-Asarone−30 group. It can be attributed to several factors such as cleavage GSH to cysteine, decrease in the synthesis of GSH and the formation of mixed disulfides, causing their cellular stores to be depleted [28].

**Table 3:** Effect of β-Asarone on Hippocampus GSH

**Different**	**Hippocampus GSH**	**F-values**	**df-values**	**p-values**
**groups**	**(nmol GSH/mg protein)**			
MCAO group	0.760 ± 0.035			
SHAM group	2.398 ± 0.013^**^	7.993	6.286	0.009
β-Asarone-10 group	1.115 ± 0.005	1.860	11	0.379
β-Asarone-20 group	2.565 ± 0.015^**^	6.536	6.400	0.008
β-Asarone-30 group	1.333 ± 0.002^*^	2.965	11	0.025

Values are shown as means ± SEM. *p <0.05 vs. MCAO group, **p < 0.01 vs. MCAO group.

**Table 4:** Effect of β-Asarone on Cerebral cortex GSH

**Different**	**Cerebral cortex GSH**	**F-values**	**df-values**	**p-values**
**groups**	**(nmol GSH/mg protein)**			
MCAO group	1.112 ± 0.010			
SHAM group	1.823 ± 0.026^*^	4.193	11	0.017
β-Asarone-10 group	1.195 ± 0.021	4.043	11	0.336
β-Asarone-20 group	1.595 ± 0.053^*^	3.306	11	0.026
β-Asarone-30 group	1.395 ± 0.011^*^	3.418	11	0.427

Values are shown as means ± SEM. *p <0.05 vs. MCAO group.

The large numbers of polyunsaturated fatty acids make cell membranes particularly vulnerable to lipid peroxidation. The oxidation of polyunsaturated fatty acids alters the structure of the membrane with resultant changes in fluidity and permeability. Lipid peroxidation can also inhibit the function of membrane bound receptors and enzymes [29]. The level of LPO content adds to the proof of the increased peroxidative damage during cerebral ischemia. A significant increase in the content of LPO was observed in the MCAO group when compared with the SHAM group. In the β-Asarone−20 and β-Asarone−30 group, a significant decrease was seen in the level of LPO when compared with the MCAO group (Table 5).

**Table 5:** Effect of β-Asarone on LPO level

**Different groups**	**nmol LPO/g**	**F-values**	**df-values**	**p-values**
	**protein**			
MCAO group	20.21 ± 1.41			
SHAM group	13.23 ± 0.66^**^	5.254	6.996	0.003
β-Asarone-10 group	20.01 ± 0.21	19.460	5.010	0.846
β-Asarone-20 group	15.32 ± 0.11^*^	1.304	10	0.043
β-Asarone-30 group	15.22 ± 0.21^*^	1.305	10	0.042

Values are shown as means ± SEM. *p <0.05 vs. MCAO group, **p < 0.01 vs. MCAO group.

It has been proposed that antioxidant changes reflect an altered redox balance in several pathological states. The antioxidants would be consumed in the reaction with free radicals. Therefore, the measurement of endogenous antioxidants enzymes i.e. GPx, GR, CAT and GST as well as Na^+^-K^+^-ATPase has been performed to estimate the amount of oxidative stress. Activities of various antioxidant enzymes and Na^+^-K^+^-ATPase of different groups have been listed in Tables 6, 7, 8, 9 and 10. The activity of endogenous antioxidant enzymes was decreased significantly in the MCAO group, as compared to the sham group, whereas in the β-Asarone−20 group, β-Asarone-treatment showed a significant restoration in the level of various enzymes as compared with MCAO group. The same results did occur in the β-Asarone−30 group.

**Table 6:** Effect of β-Asarone on the activity of GPx

** Different groups**	** GPx**	**F-values**	**df-values**	**p-values**
MCAO	8.11 ± 0.42			
SHAM	14.28 ± 1.23^**^	0.713	11	0.001
β-Asarone-10 group	9.11 ± 1.22	0.910	11	0.306
β-Asarone-20 group	12.06 ± 1.22^**^	3.556	11	0.006
β-Asarone-30 group	11.11 ± 1.21^*^	0.053	11	0.04

Values are shown as means ± SEM. *p < 0.05 vs. MCAO group, **p < 0.01 vs. MCAO group.

**Table 7:** Effect of β-Asarone on the activity of GR

**Different groups**	**GR**	**F-values**	**df-values**	**p-values**
MCAO	20.80 ± 2.10			
SHAM	35.22 ± 2.41^**^	0.953	11	0.006
β-Asarone-10 group	21.11 ± 2.12	0.03	11	0.954
β-Asarone-20 group	30.01 ± 2.12^*^	4.394	11	0.028
β-Asarone-30 group	25.22 ± 2.01^*^	4.929	11	0.048

Values are shown as means ± SEM. *p < 0.05 vs. MCAO group, **p < 0.01 vs. MCAO group.

**Table 8:** Effect of β-Asarone on the activity of GST

**Different groups**	**GST**	**F-values**	**df-values**	**p-values**
MCAO	10.17 ± 1.10			
SHAM	15.11 ± 1.20^**^	16.088	13	0.002
β-Asarone-10group	11.60 ± 1.07	9.946	10	0.680
β-Asarone-20 group	13.61 ± 1.98^**^	19.214	11	0.008
β-Asarone-30group	12.66 ± 1.80^*^	20.293	12	0.029

Values are shown as means ± SEM. *p < 0.05 vs. MCAO group, **p < 0.01 vs. MCAO group.

**Table 9:** Effect of β-Asarone on the activity of CAT

**Different groups**	**CAT**	**F-values**	**df-values**	**p-values**
MCAO	4.20 ± 0.03			
SHAM	7.10 ± 0.03^*^	7.196	12	0.010
β-Asarone-10 group	5.77 ± 0.14	3.917	10	0.076
β-Asarone-20 group	6.70 ± 0.13^*^	6.977	11	0.02
β-Asarone-30 group	6.71 ± 0.33^*^	6.858	11	0.024

Values are shown as means ± SEM. *p < 0.05 vs. MCAO group.

**Table 10:** **Effect of β-Asarone on the activity of Na**^**+**^**K**^**+**^**ATPase**

**Different groups**	**Na**^**+**^**K**^**+**^**ATPase**	**F-values**	**df-values**	**p-values**
MCAO	2.11 ± 0.23			
SHAM	4.53 ± 0.33^**^	16.966	11	0.002
β-Asarone-10group	2.00 ±0.11	0.176	11	0.684
β-Asarone-20 group	4.22 ±0.22^*^	17.700	11	0.001
β-Asarone-30group	4.16 ±0.33^*^	16.312	11	0.002

Values are shown as means ± SEM. *p < 0.05 vs. MCAO group, **p < 0.01 vs. MCAO group.

A great deal of effort has been directed toward searching for a new drug that can be used for protection of cerebral ischemia-reperfusion injury. β-Asarone was used in the prevention of cerebral ischemia in this paper. Here we showed that the β-Asarone significantly improved the neurological outcome after cerebral ischemia and reperfusion in terms of neurobehavioral function in rats. At the same time, supplementation of β-Asarone significantly boosted the defense mechanism against cerebral ischemia by increasing antioxidants activity related to lesion pathogenesis. Restoration of the antioxidant homeostasis in the brain after reperfusion may help the brain recover from ischemic injury.

## Conclusions

These experimental results suggest that complement β-Asarone is protective after cerebral ischemia in specific way. The administration of β-Asarone significantly reduced focal cerebral ischemic/reperfusion injury. The defense mechanism against cerebral ischemia was by increasing antioxidants activity related to lesion pathogenesis.

## Competing interests

The authors declare that they have no competing interests.

## Authors’ contributions

GJY and LCY: designed the experiment; YYX and CYT: conducted research and drafting of the manuscript; ZXJ: acquisition of data; analysis and interpretation of data; statistical analysis; HCL: review of the manuscript; analysis and interpretation of data. All authors read and approved the final manuscript.

## Pre-publication history

The pre-publication history for this paper can be accessed here:

http://www.biomedcentral.com/1472-6882/13/236/prepub
